# Supplementary material for: Comparison of the Three Most Commonly Used Metabolic Syndrome Definitions in the Chinese Population: A Prospective Study
Source: Metabolites. 2022 Dec 21;13(1):12. doi: 10.3390/metabo13010012 (PMC9860556; doi:10.3390/metabo13010012)
Supplement: Supplementary file 1 [file metabolites-13-00012-s001.zip › metabolites-2031217-supplementary/Supplementary files.pdf]

# Supplementary files

Table S1 Study participants before and after multiple imputation ........................2

Table S2 Association of MetS Components defined by different criteria with the risk of cardiovascular events....................................................................................3

Table S3 Comparison of AUC with different MetS definitions............................4

Figure S1 – ROC curves for IDF, revised ATPIII and JCDCG definitions as MetS diagnostic tests for cardiovascular disease ..........................................................5

Figure S2– ROC curves for IDF, revised ATPIII and JCDCG definitions as MetS diagnostic tests for stroke.......................................................................................6

Figure S3– ROC curves for IDF, revised ATPIII and JCDCG definitions as MetS diagnostic tests for coronary heart disease............................................................7

Text S2: Weights calculation in the Study ............................................................8

## Table S1 Study participants before and after multiple imputation

| | before | Multiple imputation data | P-value |
|---|---|---|---|
| | (N=20538) | (N=20888) | |
| Age (years) | 56.3±13.1 | 56.3±13.1 | 0.852 |
| Sex | | | 0.639 |
| men | 9502 (46.3%) | 9713 (46.5%) | |
| women | 11036 (53.7%) | 11175 (53.5%) | |
| Region (n%) | | | 0.918 |
| East | 8355 (40.7%) | 8478 (40.6%) | |
| Central | 8617 (42.0%) | 8804 (42.1%) | |
| West | 3566 (17.4%) | 3606 (17.3%) | |
| Area(n%) | | | 0.816 |
| urban | 9166 (44.6%) | 9347 (44.7%) | |
| rural | 11372 (55.4%) | 11541 (55.3%) | |
| Education level(n%) | | | 0.999 |
| Middle school or below | 16370 (79.7%) | 16650 (79.7%) | |
| High school or vocational school | 2872 (14.0%) | 2922 (14.0%) | |
| College and above | 1296 (6.3%) | 1316 (6.3%) | |
| Smoking status (n%) | | | 0.85 |
| No | 15808 (77.0%) | 16060 (76.9%) | |
| Yes | 4730 (23.0%) | 4828 (23.1%) | |
| Alcohol consumption (n%) | | | 0.767 |
| No | 16501 (80.3%) | 16757 (80.2%) | |
| Yes | 4037 (19.7%) | 4131 (19.8%) | |
| Total Cholesterol(mmol/L) | 4.81±0.97 | 4.82±0.97 | 0.547 |
| LDL Cholesterol (mmol/L) | 2.82±0.812 | 2.82±0.814 | 0.607 |
| BMI (kg/m2) | 24.6±3.48 | 24.6±3.48 | 0.911 |
| Family history of cardiovascular disease (n%) | | | 0.874 |
| No | 17758 (85.0%) | 17448 (85.0%) | |
| Yes | 3130 (15.0%) | 3090 (15.0%) | |

**Table S2 Association of MetS Components defined by different criteria with the risk of cardiovascular events.**

| | Revised ATP III | *P* | IDF | *P* | JCDCG | *P* |
|---|---|---|---|---|---|---|
| CVD | | | | | | |
| Central obesity | 1.20(1.04, 1.38) | 0.015 | 1.20(1.04, 1.38) | 0.015 | 1.13(0.98, 1.29) | 0.092 |
| Low HDL cholesterol | 1.08(0.93, 1.26) | 0.292 | 1.08(0.93, 1.26) | 0.323 | 1.12(0.93, 1.35) | 0.227 |
| Elevated blood pressure | 1.76(1.49, 2.07) | <0.001 | 1.76(1.49, 2.07) | <0.001 | 1.76(1.49, 2.07) | <0.001 |
| High triglycerides | 0.87(0.74, 1.02) | 0.082 | 0.87(0.74, 1.02) | 0.084 | 0.86(0.73, 1.01) | 0.068 |
| Elevated glucose | 1.23(1.08, 1.41) | 0.002 | 1.24(1.09, 1.42) | 0.001 | 1.39(1.20, 1.60) | <0.001 |
| Stroke | | | | | | |
| Central obesity | 1.15(0.96, 1.39) | 0.128 | 1.15(0.96, 1.39) | 0.129 | 1.06(0.89, 1.27) | 0.500 |
| Low HDL cholesterol | 1.11(0.92, 1.35) | 0.271 | 1.12(0.93, 1.36) | 0.239 | 1.09(0.86, 1.38) | 0.481 |
| Elevated blood pressure | 1.89(1.53, 2.35) | <0.001 | 1.89(1.53, 2.35) | <0.001 | 1.91(1.54, 2.36) | <0.001 |
| High triglycerides | 0.91(0.74, 1.11) | 0.340 | 0.91(0.74, 1.11) | 0.333 | 0.92(0.75, 1.13) | 0.439 |
| Elevated glucose | 1.26(1.07, 1.50) | 0.007 | 1.25(1.06, 1.49) | 0.009 | 1.36(1.13, 1.63) | 0.001 |
| CHD | | | | | | |
| Central obesity | 1.27(0.98, 1.65) | 0.072 | 1.27(0.98, 1.66) | 0.070 | 1.25(0.97, 1.61) | 0.080 |
| Low HDL cholesterol | 0.92(0.70, 1.22) | 0.580 | 0.90(0.68, 1.19) | 0.454 | 1.05(0.75, 1.47) | 0.778 |
| Elevated blood pressure | 1.77(1.31, 2.40) | <0.001 | 1.77(1.30, 2.40) | <0.001 | 1.75(1.29, 2.37) | <0.001 |
| High triglycerides | 0.91(0.68, 1.21) | 0.509 | 0.92(0.69, 1.22) | 0.545 | 0.86(0.64, 1.15) | 0.305 |
| Elevated glucose | 1.40(1.10, 1.79) | 0.006 | 1.41(1.11, 1.80) | 0.005 | 1.62(1.25, 2.10) | <0.001 |

HDL, high-density lipoprotein; IDF, International Diabetes Federation; Revised ATP III, the revised US National Cholesterol Education Program Adult Treatment Panel III; JCDCG, the Joint Committee for Developing Chinese Guidelines.

## Table S3 Comparison of AUC with different MetS definitions

| | Revised ATP III | IDF | JCDCG | $P^a$ |
|---|---|---|---|---|
| Total | | | | |
| CVD | 0.547(0.531-0.563) | 0.540 (0.524-0.556) | 0.545(0.530 -0.560) | <0.001 |
| Stroke | 0.552(0.531-0.573) | 0.544(0.523-0.564) | 0.543(0.523-0.562) | <0.001 |
| CHD | 0.557(0.528-0.587) | 0.547(0.518-0.575) | 0.560(0.531-0.588) | <0.001 |
| Men | | | | |
| CVD | 0.529 (0.509 -0.550) | 0.523 (0.504 -0.542) | 0.531 (0.511 -0.550) | <0.001 |
| Stroke | 0.536 (0.510 -0.563) | 0.527 (0.502 -0.552) | 0.536 (0.510 -0.561) | 0.007 |
| CHD | 0.544 (0.506 -0.581) | 0.529 (0.494 -0.564) | 0.538 (0.502 -0.573) | 0.110 |
| Women | | | | |
| CVD | 0.585 (0.560 -0.610) | 0.580 (0.555 -0.605) | 0.562 (0.538 -0.585) | <0.001 |
| Stroke | 0.586 (0.555 -0.618) | 0.582 (0.550 -0.614) | 0.549 (0.520 -0.578) | 0.003 |
| CHD | 0.595 (0.549 -0.641) | 0.593 (0.546 -0.639) | 0.588 (0.543 -0.633) | 0.455 |

AUC, Area Under Curve; MetS, metabolic syndrome; IDF International Diabetes Federation; The revised ATP III, the revised US National Cholesterol Education Program Adult Treatment Panel III; JCDCG, the Joint Committee for Developing Chinese Guidelines; CVD, cardiovascular disease; CHD, coronary heart disease.

a: AUC comparison (DeLong, DeLong and Clarke-Pearson, 1988)

**Figure S1 – ROC curves for IDF, revised ATPIII and JCDCG definitions as MetS diagnostic tests for cardiovascular disease**

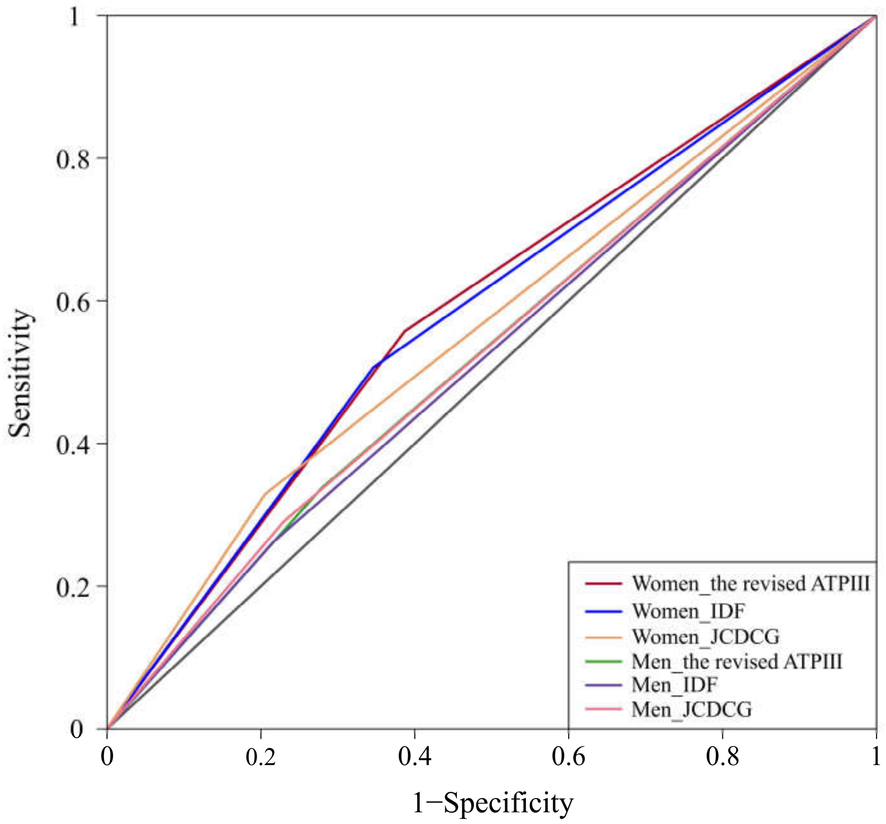


IDF International Diabetes Federation; The revised ATP III, the revised US National Cholesterol Education Program Adult Treatment Panel III; JCDCG, the Joint Committee for Developing Chinese Guidelines

**Figure S2– ROC curves for IDF, revised ATPIII and JCDCG definitions as MetS diagnostic tests for stroke**

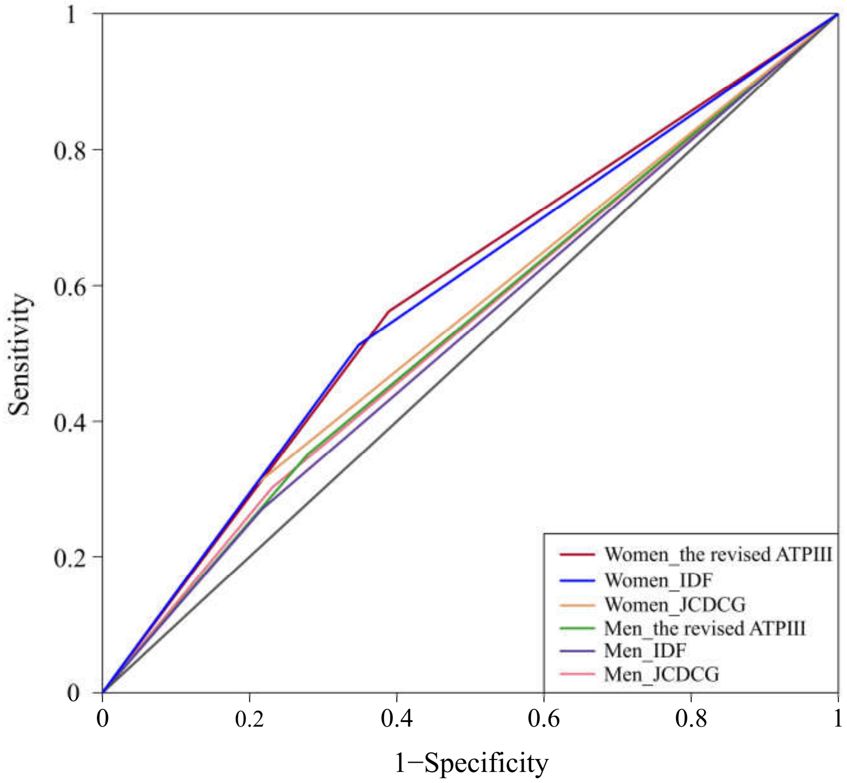


IDF International Diabetes Federation; The revised ATP III, the revised US National Cholesterol Education Program Adult Treatment Panel III; JCDCG, the Joint Committee for Developing Chinese Guidelines

**Figure S3– ROC curves for IDF, revised ATPIII and JCDCG definitions as MetS diagnostic tests for coronary heart disease.**

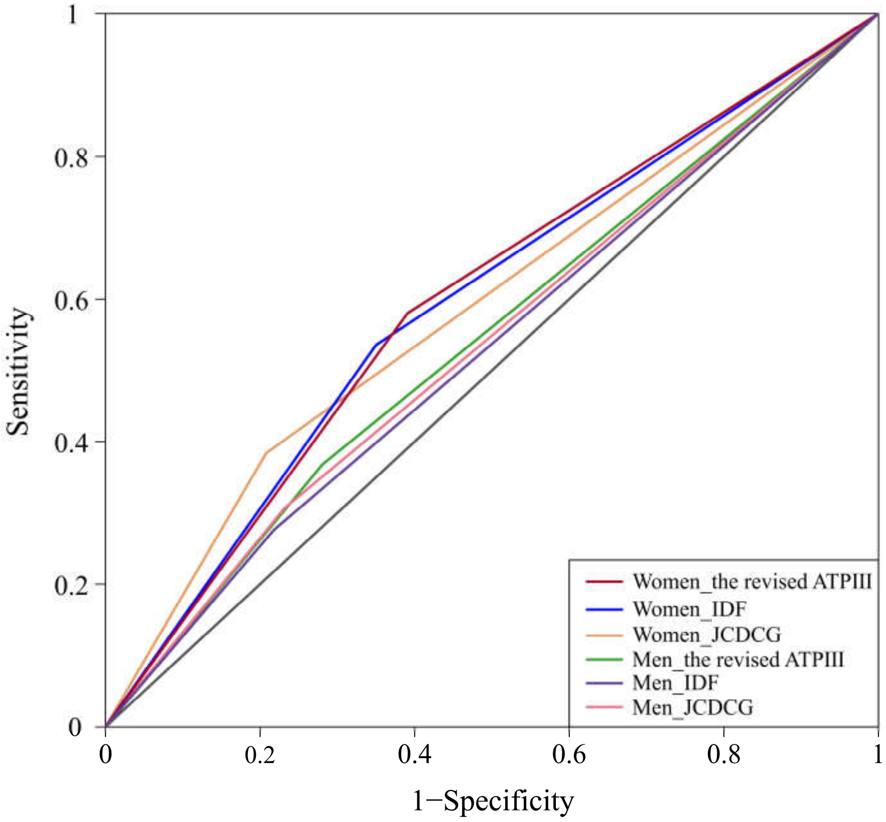


IDF International Diabetes Federation; The revised ATP III, the revised US National Cholesterol Education Program Adult Treatment Panel III; JCDCG, the Joint Committee for Developing Chinese Guidelines

## Text S2: Weights calculation in the Study

### 1. Sampling Unit Sampling Weight

The weight $w_i$ of the observed individual $i$ is the reciprocal of the individual's sampling probability, that is $w_i = 1/\pi_i$. According to the sampling design of this survey, the construction method of the basic sampling weight is as follows:

**The first stage:**

The sampling weight of sample city/county in each province is the reciprocal of sampling probability of sample city/county under stratified PPS sampling. Urban and rural stratification needs to be calculated separately. The calculation formula is as follows:

$$w_1 = \frac{\text{Urban/rural population in the provinces where the individual i located}}{\text{Population size of city/county where the individual i located *4}}$$

**The second stage:**

$w_{2|1}$ is the sampling weight of the sample district/township, and its value is the reciprocal of the simple random sampling probability of the district/township. Its calculation formula is as follows:

$$w_{2|1} = \frac{\begin{array}{l}\text{Total number of the district/town in the city/county}\\ \text{where the individual i located}\end{array}}{\begin{array}{l}\text{Sample number of the district/town in the city/county}\\ \text{where the individual i located}\end{array}}$$

**The third stage:**

$w_{3|2,1}$ is the sampling weight of sample communities/villages, and its value is the reciprocal of simple random sampling probability of communities/villages. The calculation formula is as follows:

$$w_{3|2,1} = \frac{\begin{array}{l}\text{Total number of the community/village}\\ \text{in the district/town where the individuals i located}\end{array}}{\begin{array}{l}\text{Sample number of the community/village}\\ \text{in the district/town where the individuals i located}\end{array}}$$

**The fourth stage:**

$w_{4|3,2,1}$ is the sampling weight of the sample individual, and its value is the reciprocal of the simple random sampling probability of the sample individual. The weight of this stage should be calculated by sex and age, and the formula is as follows:

$$w_{4|3,2,1} = \frac{\begin{array}{l}\text{Total number of male/female population in a certain age group of}\\ \text{the community/village where individual i located}\end{array}}{\begin{array}{l}\text{Sampling number of male/female population in a certain age group of}\\ \text{the community/village where individual i located}\end{array}}$$

According to the sampling weights of the above stages, the basic sampling weights of individual

samples are as follows:

$$w_{base} = w_1 \times w_{2|1} \times w_{3|2,1} \times w_{4|3,2,1}$$

**2. Non-response Adjustment Weight**

Individuals who make no response and individuals who participated in the survey but were missing in key variables are treated as non-responder. The population structure of the missing population was adjusted according to sex and age group of one year per 10 years old. The method is shown in the below Table ST1.

Table ST1 Construction of Adjustment Weight for Non-response

| Sex | Age group (years) | | | |
|---|---|---|---|---|
| | 15～24 | 25～34 | … | ≥75 |
| Men | $\sum_{i=1}^{n_{11}} w_i \Big/ \sum_{i=1}^{n'_{11}} w_i$ | $\sum_{i=1}^{n_{12}} w_i \Big/ \sum_{i=1}^{n'_{12}} w_i$ | $\sum_{i=1}^{n_{1(c-1)}} w_i \Big/ \sum_{i=1}^{n'_{1(c-1)}} w_i$ | $\sum_{i=1}^{n_{1c}} w_i \Big/ \sum_{i=1}^{n'_{1c}} w_i$ |
| Women | $\sum_{i=1}^{n_{21}} w_i \Big/ \sum_{i=1}^{n'_{21}} w_i$ | $\sum_{i=1}^{n_{22}} w_i \Big/ \sum_{i=1}^{n'_{22}} w_i$ | $\sum_{i=1}^{n_{2(c-1)}} w_i \Big/ \sum_{i=1}^{n'_{2(c-1)}} w_i$ | $\sum_{i=1}^{n_{2c}} w_i \Big/ \sum_{i=1}^{n'_{2c}} w_i$ |

$$w'_{adj} = \frac{\sum_{i=1}^{n_{rc}} w_i}{\sum_{i=1}^{n'_{rc}} w_i}$$

$\sum_{i=1}^{n_{rc}} w_i$ is the sum of the basic sampling weights of all the people who fall in row R and column C, $\sum_{i=1}^{n'_{rc}} w_i$ is the sum of the basic sampling weights of all respondents in the sample population who fall in line R and in line C.

**3. Demographic Adjustment Weight**

Overall, the total population over 18 years old in 2010 was used. The data came from the 2010 National Bureau of Statistics census. The sum of the basic weights of all observed individuals in the sample is the estimated total number of people over 18 years old in 31 provinces (autonomous regions or municipalities). The adjustment method is shown in Table ST2.11.

Table ST2 Construction of Demographic Adjustment Weight

| Sex | Age group (years) | | | |
|---|---|---|---|---|
| | 18-24 | 25-34 | … | ≥75 |
| Men | $N_{11} \Big/ \sum_{i=1}^{n_{11}} w_i$ | $N_{12} \Big/ \sum_{i=1}^{n_{12}} w_i$ | $N_{1(c-1)} \Big/ \sum_{i=1}^{n_{1(c-1)}} w_i$ | $N_{1c} \Big/ \sum_{i=1}^{n_{1c}} w_i$ |
| Women | $N_{21} \Big/ \sum_{i=1}^{n_{21}} w_i$ | $N_{22} \Big/ \sum_{i=1}^{n_{22}} w_i$ | $N_{2(c-1)} \Big/ \sum_{i=1}^{n_{2(c-1)}} w_i$ | $N_{2c} \Big/ \sum_{i=1}^{n_{2c}} w_i$ |

$$w_{adj} = \frac{N_{rc}}{\sum_{i=1}^{n_{rc}} w_i}$$

$N_{rc}$ is the number of natural population in line R and column C, $\sum_{i=1}^{n_{rc}} w_i$ is the sum of basic sampling weights of all sample population in line R and column C.

**4. Survey Design Weight**

The weights of survey design for observing individuals are as follows:

$$w_i = w_1 \times w_{2|1} \times w_{3|2,1} \times w_{4|3,2,1} \times w'_{adj} \times w_{adj}$$
